# Undiagnosed Primary Hyperparathyroidism and Recurrent Miscarriage: The First Prospective Pilot Study

**DOI:** 10.1007/s00268-017-4395-7

**Published:** 2018-01-18

**Authors:** Aimee DiMarco, Ioannis Christakis, Vasilis Constantinides, Lesley Regan, F. Fausto Palazzo

**Affiliations:** 1 Department of Endocrine and Thyroid Surgery, Hammersmith Hospital, Imperial College London, London, UK; 2 Department of Gynaecology, Imperial College London, London, UK

## Abstract

**Background:**

Primary hyperparathyroidism (pHPT) in pregnancy is reported to be associated with significant maternal and foetal complications and an up to threefold increase in the risk of miscarriage. However, the true incidence of pHPT in pregnancy, complete and miscarried, is unknown and there are no data on the prevalence of undiagnosed pHPT in recurrent miscarriage (RM) (≥3 consecutive miscarriages under 24-week gestation). This is the first prospective study aiming to establish the prevalence of undiagnosed pHPT in RM.

**Methods:**

Following UK National ethics committee approval, women who had experienced 3 or more consecutive miscarriages were recruited from a nationwide RM clinic. Serum corrected calcium, phosphate, PTH and vitamin D were evaluated. Patients with raised serum calcium and/or PTH were recalled for confirmatory tests. Power calculations suggested that a minimum of 272 patients were required to demonstrate a clinically significant incidence of pHPT.

**Results:**

Three hundred women were recruited, median age 35 years (range 19–42). Eleven patients had incomplete data, leaving 289 patients suitable for analysis; 50/289 patients (17%) with abnormal tests were recalled. The prevalence of vitamin D deficiency (<25 nmol/l) and insufficiency (25–75 nmol/l) was 8.7 and 67.8%, respectively. One patient was diagnosed with pHPT (0.34%) and underwent successful parathyroidectomy.

**Conclusions:**

The prevalence of undiagnosed pHPT (0.34%) in RM in this study appears to be many times greater than the 0.05% expected in this age group. The findings of this pilot study merit follow-up with a larger-scale study. Routine serum calcium estimation is not currently undertaken in RM and should be considered.

## Introduction

Primary hyperparathyroidism (pHPT) is the third commonest endocrine disorder after diabetes and thyroid disease [1–3], with a prevalence of 0.15–0.4% in the general population and twofold female preponderance [2, 4]. The majority of patients with pHPT are diagnosed in late middle age onwards, with less than 1% currently diagnosed during pregnancy [3–5]. However, serum calcium is not routinely measured in pregnancy so the true incidence of pHPT in the gravid woman, complete and miscarried, is unknown.

Miscarriage is defined as the loss of a foetus at any time from conception to week 24 of pregnancy and recurrent miscarriage, according to WHO guidelines, three or more consecutive miscarriages before week 24 [6]. The largest surgical case series of pHPT in pregnancy reports a threefold risk of miscarriage, with the rate of foetal loss progressively increasing with maternal serum calcium [5]. However, this study is likely to suffer from sampling bias, the methodology having been enquiry about a prior history of miscarriage in patients already diagnosed with pHPT and selected for parathyroidectomy. Larger retrospective cohort studies from registry data in Israel [7] and Denmark [1] do not, at face value, appear to corroborate the findings of this study.

RM affects 1% of couples trying to conceive and results in considerable psychological burden. Despite this, it has historically been a neglected area of research, and as a result, the causes are not well understood. Our hospital runs the UK’s largest specialist tertiary referral clinic for women with RM, receiving referrals from all over the UK and beyond. At present, investigations for potential causes of RM do not include screening for pHPT with serum calcium estimation, despite the fact that it is possible that this is contributory and reversible with parathyroidectomy. There has been no previous prospective data on the prevalence of undiagnosed pHPT in women experiencing consecutive recurrent miscarriage (RM). This prospective pilot study was therefore devised to establish the prevalence of undiagnosed pHPT in RM patients and assess whether routine screening for pHPT in this population may be justified.

## Materials and methods

**Fig. 1:**
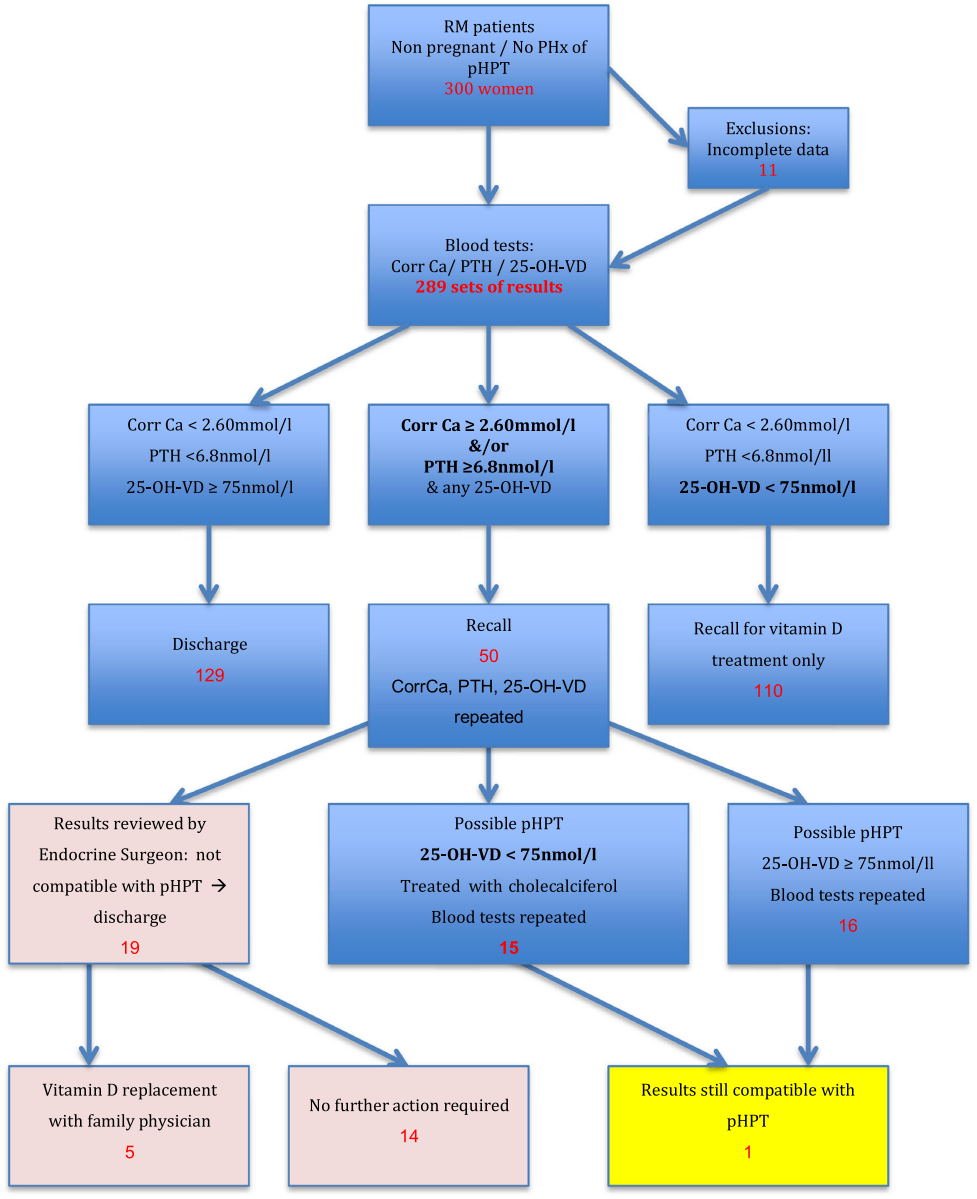
Patient flow through the study. *f/u* follow-up, *25*-*OH*-*VD* 25-hydroxy vitamin D, *PTH* parathyroid hormone, *pHPT* primary hyperparathyroidism

Following national ethics committee approval, recruitment was undertaken in our hospital’s RM clinic, of women who had experienced 3 or more consecutive miscarriages at under 24 weeks. Exclusion criteria were failure to meet the definition of RM, previous parathyroid or thyroid surgery, a known MEN syndrome, lithium use and current pregnancy. Figure 1 shows patient flow through the study. Serum corrected calcium, phosphate, PTH and vitamin D were evaluated. Elevated serum calcium was defined as an albumin-corrected value >2.6 mmol and PTH >6.8 pmol/l. Vitamin D deficiency was defined as a 25-hydroxyvitamin D of <25 nmol/l, insufficiency as 25–75 nmol/l and adequacy ≥75 nmol/l.

Patients with vitamin D deficiency or insufficiency in isolation were treated with vitamin D supplements, and serum calcium, PTH and vitamin D were rechecked following treatment to confirm normalization of the biochemistry. Ongoing care was then transferred back to the RM clinic and general practitioner. Patients with a raised serum calcium and/or PTH had their tests repeated and were recalled for review by a consultant endocrine surgeon (FP). At recall, those with results excluding pHPT were discharged to their general practitioner and RM clinic. Patients with results potentially still compatible with pHPT and with coexistent vitamin D deficiency or insufficiency were treated with cholecalciferol 20,000 units twice weekly for 6 weeks and their biochemical profile repeated. Persistently abnormal results prompted further investigation for possible primary hyperparathyroidism and a follow-up visit.

Parathyroid hormone in the study patients was measured using automated immunoassays (Abbott Architect and Immulite 2000, Siemans, Llanberis, UK). 25-Hydroxyvitamin D was measured using the automated liaison assay (Diasorin, Saluggia, Italy). Coefficient of variation was <8% and <11%, respectively, across the diagnostic range [8].

At the time the study was designed, the best available data suggested a background prevalence of pHPT in women of reproductive age of 36/100,000 [9]. The required sample size was calculated using the STATA corporation statistical platform [10, 11] and based on detecting a change in prevalence from 0.036% (i.e. 36/100,000) in the general population to 1% or more in the RM population. One per cent prevalence for pHPT in the RM group was chosen as it is generally regarded as the lowest value that may stimulate a change in clinical practice. A one-tailed test, with a power of 80% (i.e. *β* = 0.2) and significance level (*α*) of 5%, showed a minimum requirement of 272 RM patients with binomial 95% confidence intervals around the 1% prevalence of 0.23–3.19%. Descriptive statistics were calculated in Excel^®^ (V14.6, 2010, Microsoft^®^, Washington, USA).

## Results

Three hundred women with a median age of 35 years (19–42) were recruited from the RM clinic over a 2-year period. Figure 1 shows their progress through the study. All patients were eligible for inclusion; none met the exclusion criteria. Eleven patients who had incomplete data, mainly due to failure to attend for initial or follow-up investigations and after attempts to contact them directly and via their general practitioner, were excluded from the study leaving 289 women. Table 1 shows their results: median serum corrected calcium was 2.38 mmol/l (range 2.09–2.71 mmol/l) and PTH 5.2 (range 1.3–18.4). Median vitamin D level was 53.8 nmol/l (range 6.4–134 nmol/l) with a prevalence of vitamin D deficiency (25-hydroxyvitamin <25 nmol/l) and insufficiency (25–75 nmol/l) in the whole cohort of 8.7% and 67.8%, respectively. Only 22.8% were vitamin D replete (≥75 nmol/l).

**Table 1:** Demographics and biochemical parameters of the study participants

Variable	Median	Range	pHPT patient (values at initial review and pre-op)
Age at enrolment (years)	35	19–42	34
Corrected calcium (mmol/l)	2.38	2.09–2.71	2.60, 2.64
PTH (pmol/ml)	5.2	1.3–18.4	18.4, 13.4
25-OH-vitamin D (nmol/l)	53.8	6.4–134	31, 102

Normal ranges, corrected calcium 2.20–2.60 mmol/l, PTH 1.1–6.8 pmol/ml, 25-OH-vitamin D 75–150 nmol/l

**Fig. 2:**
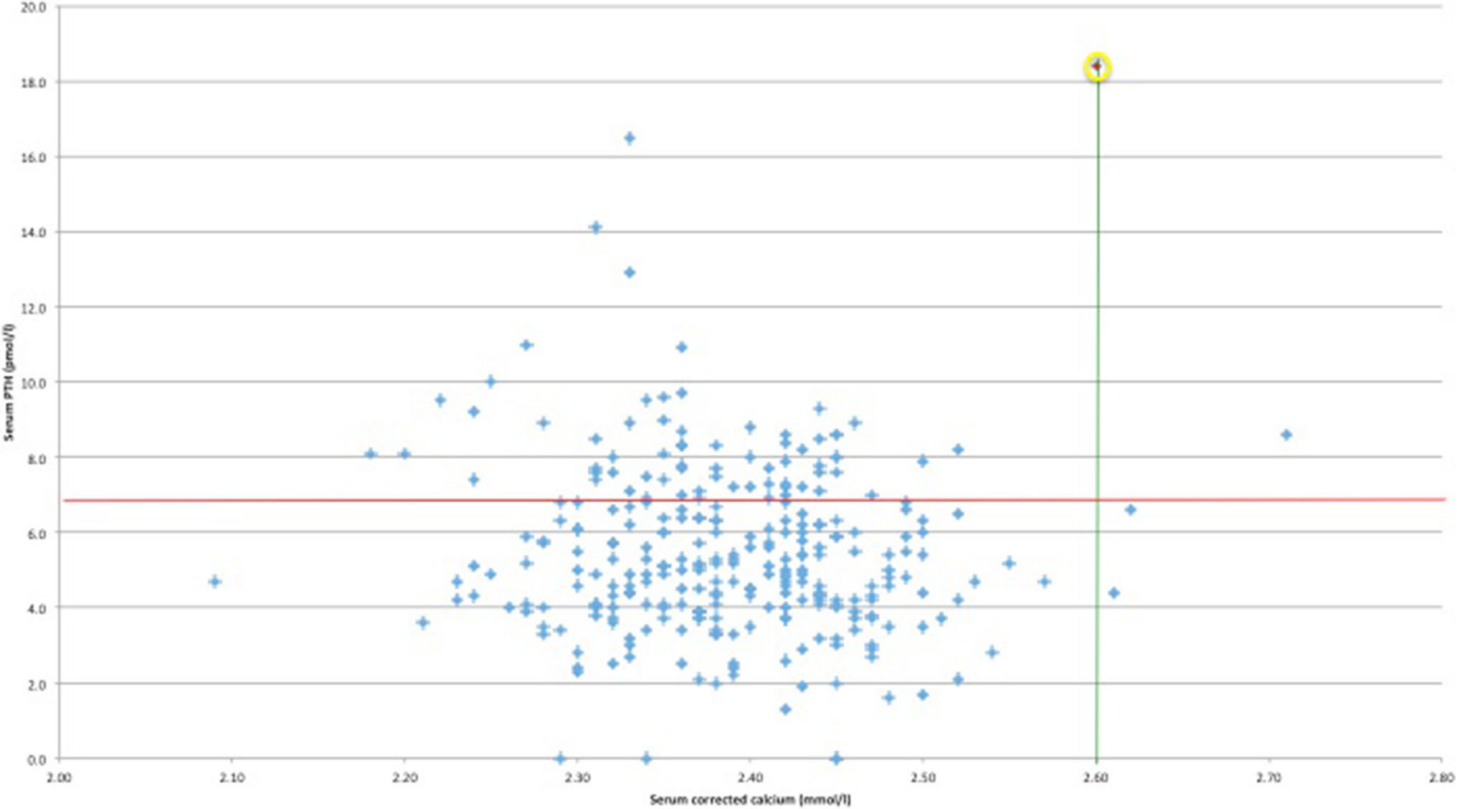
Serum PTH (y-axis) vs corrected calcium (x-axis) for every patient. Upper limits of normal are shown in red (serum PTH > 6.8 pmol/l) and green (corrected calcium > 2.6 mmol/l). Single patient with phHPT in yellow circle

Fifty patients (17%) were recalled due to results suspicious for pHPT (Fig. 2). In addition, 110 (38%) were found to have inadequate levels of vitamin D in isolation (<75 nmol/l). These women were commenced on supplements and after normalization of their biochemistry returned to standard care in the RM clinic. Of the 50 recalled patients, 19 had a diagnosis of pHPT excluded at their recall appointment and 31 were investigated further: Fifteen of those required vitamin D supplementation, the others undergoing repeat biochemical testing of blood and urine alone. Ultimately, one patient had results that, after vitamin D supplementation and assessment of the urinary calcium excretion, confirmed pHPT. This patient, aged 34, had a history of 3 previous consecutive miscarriages between 2- and 9-weeks gestation with no successful pregnancies. Her other past medical history was of migraine and chest pain, for which she had consulted her GP and attended the emergency department, with no cause elucidated. She had had no renal calculi nor fragility fractures. Her bone density was unknown. There was no family history of endocrine disease nor features suggestive of a genetic syndrome. Corrected calcium at presentation was 2.6 mmol/l, PTH 18.4 pmol/ml, phosphate 0.92 mmol/l and 25-hydroxyvitamin D 31 nmol/l. Vitamin D supplementation was instituted as described above. This improved the serum 25-hydroxyvitamin D (to 102 nmol/l), but the calcium remained elevated at 2.64 mmol/l and PTH at 13.4 pmol/ml. Urinary calcium concentration was 2.83 mmol/l, totalling 4.95 mmol over 24 h, and calcium/creatinine clearance ratio was 1.52% [12]. Given her urinary calcium/creatinine clearance ratio, age of 34 and absence of a family history, she did not fulfil our departmental criteria for genetic testing for either FHH or MEN and so went on to have localization studies in the form of a neck ultrasound and SestaMIBI with SPECT. These showed only a multinodular goitre and no areas of abnormal tracer uptake or retention. Following discussion with the patient and after obtaining full, informed, written consent, parathyroidectomy was performed. A bilateral neck exploration was undertaken under general anaesthesia via a standard cervicotomy.

At operation, the thyroid gland was nodular but not significantly enlarged, and four parathyroid glands were found in eutopic positions, with a single, abnormal left superior parathyroid adenoma, which was removed. Recurrent laryngeal nerves were identified and preserved with good signal on the neural monitor (NIM^®^, Medtronic™, USA). Intra-operative PTH (STAT IO-I-PTH, Future Diagnostics Solutions B.V., Wijchen, Netherlands) fell to less than 50% of the maximal value by 10 min after excision of the abnormal parathyroid gland [13]. Histopathology confirmed a parathyroid adenoma composed predominantly of oxyphil cells and measuring 18 × 17 × 3 mm, weight 0.44 g. Fibreoptic nasendoscopy performed pre- and post-operatively showed normal vocal cord function. At two-week follow-up, the patient was well with no complications and biochemical cure was confirmed with a corrected serum calcium of 2.22 mmol/l and PTH of 2.3 pmol/l. At six-month follow-up, the patient’s biochemistry remained normal. She had not yet attempted to conceive but was planning to start in autumn 2017.

The prevalence of confirmed pHPT in this cohort of patients with RM was therefore 1 in 289, i.e. 0.34%.

## Discussion

The aim of this study was to ascertain the incidence of pHPT in a population with RM in order to move closer to understanding whether there is causality. The study was designed prospectively, the first of its kind, as a pilot, whose results would inform a future larger trial, if justified. One significant difficulty in establishing whether the incidence of 0.34% in RM is of clinical relevance is the lack of a high-quality control group of non-pregnant women of childbearing age, or of pregnant women not experiencing RM. Until recently, the majority of the published literature on pHPT in women of childbearing age focused on individual cases of pHPT in pregnant women with case reports and small series, covering 119 patients between 1998 and 2017 and reporting mostly on the complications of pHPT in pregnancy including foetal loss in 37 cases [14–29].

One case series of women undergoing parathyroidectomy over a 6 year period at a single centre in Florida reported a prevalence of pHPT in women of reproductive age (16–44 years) of 8% (360 of 4500 women). This improbably high prevalence is likely to reflect referral bias to this high-volume specialist centre. Data derived from hospital admissions in Switzerland showed an in-hospital prevalence of pHPT totalling 36.3 per 100,000 women (0.04%) aged 15 to 39 years [9]. Potentially the largest study of all, a Danish registry study of women aged 16–44 years reported 1057 cases of pHPT (based on ICD8 and ICD10 codes) amongst all patients registered in the ‘National Hospital Discharge Register, LPR’ in the 33 years from January 1977 to December 2010, but did not state a denominator, preventing calculation of the prevalence [1]. The largest and probably most reliable study to report a prevalence was based on serum biochemistry, rather than hospital coding, derived from data held by a large Israeli healthcare organization: the serum calcium and PTH testing performed on 292,024 Israeli women aged 20–40 retrospectively identified 159 women, equating to 0.05% with pHPT.

The risk of foetal loss in pregnant women with pHPT is, according to the retrospective surgical series of 32 pregnant women undergoing parathyroidectomy, elevated threefold [5]. This study also showed a correlation between increasing maternal serum calcium and the rate of foetal loss with the percentage of pregnancies ending in miscarriage rising progressively from 12.5% at a serum calcium of 2.59–2.67 mmol/l to 80% at 3.04–3.24 mmol/l. This would seem to strengthen the argument that there is a causative association between the biochemical severity of pHPT and miscarriage. Indeed, although the Israeli study [7] did not show a significant difference in miscarriage between their pHPT (*n* = 74) and control groups, it was characterized by a very mild median hypercalcaemia of 2.67 mmol/l (range 2.59–3.17 mmol/l) corresponding to the mildest subgroup within the 32 patient surgical series [5]. The single hyperparathyroid patient in our study also had a mild hypercalcaemia at 2.64 mmol/l. The Danish registry study [1] showed a non-significant increase in the risk of abortion in the year after diagnosis with pHPT (relative risk = 1.5, 95% confidence interval 0.81–2.74) and those women who underwent surgery for their pHPT had a higher rate of stillbirth before the time of diagnosis of pHPT compared to those with pHPT who did not undergo surgery. This may also reflect a relationship between disease severity and risk of pregnancy loss; however, serum calcium was not reported in this study.

The existing data therefore appear to be statistically insufficient to exclude a genuine nexus between pHPT in patients with RM and are further complicated by the difficulty in retrospective studies in establishing the time of onset of pHPT in relation to pregnancy and miscarriage. To this, one should add the possible underestimation in the incidence of pHPT in women who do become pregnant due to the overlap in non-specific symptoms associated with pHPT and pregnancy and the diagnostic challenge caused by the physiological changes of pregnancy that may mask the biochemical features of pHPT.

It should be emphasized that the population in our study group refers specifically to women who have experienced three or more consecutive miscarriages in whom no other aetiology has been identified. This represents approximately 1% of all couples trying to conceive [30]. The likelihood of this event occurring along with pHPT in a single woman of reproductive age, using the Israeli cohort of 0.05% [1], without causality and completely by chance could therefore be estimated at 0.0005%, nearly 700-fold less than the result of our study.

On a side note, the prevalence of vitamin D deficiency and insufficiency of 8.7 and 67.8% respectively in this study is in keeping with previous studies which range from 5–50% depending upon the definition and the ethnic mix of the population, with lower levels being more prevalent in black and Asian women [31, 32]. Vitamin D deficiency may result in maternal osteomalacia, myopathy and craniotabes as well as neonatal rickets and possibly intrauterine growth retardation [33, 34], but importantly it is not an independent risk factor for miscarriage. One reason for measuring and correcting vitamin D in this study group was to ensure that cases of normocalcaemic hyperparathyroidism, masked by vitamin D insufficiency, were not missed.

The optimal management of pHPT in women with RM in the non-pregnant state is parathyroid surgery, in accordance with NIH guidelines [35]. When pHPT is diagnosed in pregnancy, the treatment requires an assessment of the risks to mother and foetus of parathyroid surgery against the risks to both of postponing surgical treatment and issues of localization studies, i.e. the risk of radiation exposure to the foetus if SestaMIBI scanning is used must be considered

In conclusion, this is the first prospective study of pHPT in RM patients. The prevalence of undiagnosed pHPT of 0.34% in RM appears to be many times higher than expected in this age group according to the published literature. This pilot study justifies further study of the potentially important relationship between pHPT and RM with a larger sample size. At present in the UK, women experiencing RM do not undergo routine serum calcium estimation. Given the distress caused by RM versus the low cost of screening and the potential benefits of identifying a potentially reversible cause with a curative parathyroidectomy, it appears that routine serum calcium and PTH estimation is justified in the RM population.
